# Ethanol Induces Microglial Cell Death via the NOX/ROS/PARP/TRPM2 Signalling Pathway

**DOI:** 10.3390/antiox9121253

**Published:** 2020-12-09

**Authors:** Muhammad Syahreel Azhad Sha’fie, Sharani Rathakrishnan, Iffa Nadhira Hazanol, Mohd Haziq Izzazuddin Dali, Mohd Ezuan Khayat, Syahida Ahmad, Yazmin Hussin, Noorjahan Banu Alitheen, Lin-Hua Jiang, Sharifah Alawieyah Syed Mortadza

**Affiliations:** 1 Department of Biochemistry, Faculty of Biotechnology and Biomolecular Sciences, Universiti Putra Malaysia, Serdang 43400, Selangor, Malaysia; 188825@student.upm.edu.my (M.S.A.S.); gs57745@student.upm.edu.my (S.R.); 190382@student.upm.edu.my (I.N.H.); 189944@student.upm.edu.my (M.H.I.D.); m_ezuan@upm.edu.my (M.E.K.); syahida@upm.edu.my (S.A.); 2 Department of Cell and Molecular Biology, Faculty of Biotechnology and Biomolecular Sciences, Universiti Putra Malaysia, Serdang 43400, Selangor, Malaysia; yazminh93@gmail.com (Y.H.); noorjahan@upm.edu.my (N.B.A.); 3 Sino-UK Joint Laboratory of Brain Function and Injury of Henan Province and Department of Physiology and Pathophysiology, Xinxiang Medical University, Xinxiang 453003, China; 4 School of Biomedical Sciences, Faculty of Biological Sciences, University of Leeds, Leeds LS2 9JT, UK

**Keywords:** alcoholism, microglia, cell death, ROS, oxidative stress, PARP, TRPM2, NOX

## Abstract

Microglial cells are the primary immune cell resident in the brain. Growing evidence indicates that microglial cells play a prominent role in alcohol-induced brain pathologies. However, alcohol-induced effects on microglial cells and the underlying mechanisms are not fully understood, and evidence exists to support generation of oxidative stress due to NADPH oxidases (NOX_-mediated production of reactive oxygen species (ROS). Here, we investigated the role of the oxidative stress-sensitive Ca^2+^-permeable transient receptor potential melastatin-related 2 (TRPM2) channel in ethanol (EtOH)-induced microglial cell death using BV2 microglial cells. Like H_2_O_2_, exposure to EtOH induced concentration-dependent cell death, assessed using a propidium iodide assay. H_2_O_2_/EtOH-induced cell death was inhibited by treatment with TRPM2 channel inhibitors and also treatment with poly(ADP-ribose) polymerase (PARP) inhibitors, demonstrating the critical role of PARP and the TRPM2 channel in EtOH-induced cell death. Exposure to EtOH, as expected, led to an increase in ROS production, shown using imaging of 2’,7’-dichlorofluorescein fluorescence. Consistently, EtOH-induced microglial cell death was suppressed by inhibition of NADPH oxidase (NOX) as well as inhibition of protein kinase C. Taken together, our results suggest that exposure to high doses of ethanol can induce microglial cell death via the NOX/ROS/PARP/TRPM2 signaling pathway, providing novel and potentially important insights into alcohol-induced brain pathologies.

## 1. Introduction

Alcoholism represents a major cause of morbidity and mortality worldwide, as reported by the National Institute of Alcohol Abuse and Alcoholism, as part of the US National Institutes of Health [1]. Excessive alcohol consumption has numerous detrimental effects on many parts of the human body, leading to liver diseases, heart diseases, and neuropathologies such as alcohol use disorder, a severe and relapsing mental health disease due to alcohol-induced alterations of brain functions [1,2,3,4,5,6,7]. In addition, as has been well documented, alcohol intake causes the brain to promote further alcohol consumption [7]. Regarding how alcohol impacts the brain, multiple molecular and cellular mechanisms have been proposed, including increased poly(ADP-ribose) polymerase (PARP) activity and ensuing regulation of gene expression, induction of oxidative stress via NADPH oxidases (NOX)-mediated generation of reactive oxygen species (ROS), neurotoxicity, neuromodulation, and neuroinflammation [7,8,9,10,11,12].

Microglial cells are the privileged immune cells in the central nervous system (CNS) and they play a central role in innate immune signaling and are vital for the surveillance and maintenance of the homeostasis in the CNS [13,14,15]. However, it is well recognized that chronic or dysregulated microglial activation can cause excessive generation of proinflammatory cytotoxic mediators, including ROS [13,14,15]. Microglial cells are known to be activated by structurally diverse molecules, including ethanol (EtOH). Increasing attention has been devoted to understanding the role of microglial cells in alcohol-induced signaling, and compelling evidence has been gathered to support the conclusion that microglial cell-mediated inflammatory responses are important in mediating alcohol-induced toxicity in both fetal and adult brains [7,11,16,17,18,19,20,21,22,23,24]. More specifically, several molecular mechanisms have been identified in alcohol-induced microglial cell-mediated neuroinflammation, including NOX, which mediates ROS generation, and ATP-sensitive P2X7 receptor, which is crucially involved in the generation of proinflammatory cytokines and neuroinflammation [11,24]. It is well recognized that exposure to high amounts of alcohol can cause neuronal cell death, which is critical in contributing to alcohol-related brain damage, but the effect of alcohol on microglial cell viability is unknown [11,12,18]. However, there is increasing evidence to show microglial cells’ demise in response to oxidative stress induced by exposure to ROS or ROS-inducing pathological factors [25,26].

Transient receptor potential melastatin-related 2 (TRPM2) is a Ca^2+^-permeable cationic channel gated by intracellular ADP-ribose (ADPR) [27]. The TRPM2 channel is highly sensitive to activation by high levels of ROS or oxidative stress, as a result of ADPR generation via engaging poly(ADPR) polymerases (PARP), particularly PARP-1, in the nucleus [27]. Now, there is a large volume of experimental evidence to support the TRPM2 channel as one of the critical molecular mechanisms for oxidative stress-related pathologies [28,29]. The TRPM2 channel is known to be highly expressed in microglial cells [25,26,30,31,32,33]. Recent studies from our group and other authors using pharmacological and/or genetic interventions have shown an important role for the TRPM2 channel in signaling mechanisms mediating the responses of microglial cells, including cell death, to ROS and pathological factors, such as amyloid β-peptide, MPP^+^, and Zn^2+^, which are known to be able to stimulate ROS generation [25,26,31]. In this study, we are interested in whether alcohol-induced oxidative stress results in microglial cell death and, if this is true, whether and how the TRPM2 channel is involved in such alcohol-induced cell death, using murine BV2 microglial cells, a widely-used cell model for in vitro studies of microglial cell-mediated mechanisms, including those induced by ethanol [34,35]. We show that exposure to high doses of EtOH induces microglial cell death and provide evidence to show that EtOH causes microglial cell death via induction of NOX-mediated ROS generation and subsequent activation of PARP and the TRPM2 channel. Our findings have led us to propose a novel mechanism that is potentially critical in mediating alcohol-induced brain pathologies.

## 2. Materials and Methods

### 2.1. Chemicals and Reagents

All chemicals or reagents were commercially obtained from Sigma-Aldrich unless specified otherwise. PJ34 was from Santa Cruz, GKT137831 from Cayman Chemical, and chelerythrine chloride (CTC) from Tocris.

### 2.2. BV2 Cell Culture and Preparation

BV2 cells, a mouse microglia cell line ICLCATL03001 originally from Cell Line Collection Banca Biologica e Cell Factory, were maintained in Dulbecco’s modified Eagle medium (DMEM) containing 25 mM glucose, supplemented with 10% heat-inactivated fetal bovine serum (FBS) and maintained in a tissue culture incubator at 37 °C under 5% CO_2_ humidified conditions. Cells were cultured in 25-cm^2^ flasks and passaged when cells reached >80% confluence. Cells were detached from the bottoms of flasks using 0.05% trypsin-EDTA (Invitrogen), collected by centrifugation, re-suspended in fresh culture media, and sub-cultured in 25-cm^2^ flasks or in 96-well plates for experimentation.

### 2.3. Reverse Transcription-Polymerase Chain Reaction (RT-PCR)

Total RNA was isolated using the TRIZOL reagent (Invitrogen) and dissolved into RNase-free water, and first-strand cDNA was synthesized using SuperScript II reverse transcriptase (Invitrogen) according to the manufacturer’s instructions. PCR was conducted in 20 μL using Mastercycler 5333 (Eppendorf, Hamburg, Germany) and Taq DNA polymerase (New England BioLabs). The forward and reverse primer sequences are 5′-AAGCCTAAGTGTCCTGAGAGCG and 5′-ATGTCCAGCAGATCCACCATGG, with the PCR product having a size of 479 bp. The PCR conditions used were as follows: initial denaturation at 95 °C for 2 min followed by 35 cycles of 95 °C for 30 s, 59 °C for 60 s, and 72 °C for 30 s. The PCR products were resolved in 2% agarose gels and documented using a Gel Imaging and Quantity One (Biorad).

### 2.4. Immunofluorescent Staining

Cells were seeded on 13-mm glass coverslips 12–24 h before use. Cells were rinsed with phosphate buffer saline (PBS: 8 g/L NaCl, 0.2 g/L KCl, 1.44 g/L Na_2_PO_4_, and 0.24 g/L KH_2_PO_4_ in water, pH 7.4) and fixed using methanol for 5 min at −20 °C. After washing with PBS containing 0.4% (*v:v*) Trition X-100 (PBS-T), cells were incubated in the blocking solution (PBS with 10% (*w:v*) bovine serum albumin) at room temperature for 30 min. Cells were incubated with the primary rabbit anti-TRPM2 antibody (Bethyl) at a dilution of 1:800 at 4 °C overnight. After washing with PBS-T, cells were incubated with the secondary fluorescein isothiocyanate-conjugated anti-rabbit IgG antibody (Sigma) at 1:500 for 1 h at room temperature. After washing with PBS-T, coverslips were dried on tissue papers and mounted inversely on glass slides with anti-fade fluorescent mounting medium containing 4’,6-diamidino-2-phenylindole (DAPI) (Molecular Probes). Cells stained with only the secondary antibody were used as a negative control. Fluorescent images were captured using a LSM510 META confocal microscope and analyzed using LSM Image (Zeiss, Jena, Germany).

### 2.5. Single-Cell Ca^2+^ Imaging

Single-cell Ca^2+^ imaging was performed at room temperature as described in our previous study [36]. In brief, cells were seeded on 13-mm glass coverslips 12–24 h before use. Cells were incubated for 1 h with 4 μM Fura-2 AM (Molecular Probes) in extracellular Ca^2+^-containing solution (134 mM NaCl, 5 mM KCl, 1.2 mM MgCl_2_, 1.5 mM CaCl_2_, 8 mM glucose, and 10 mM HEPES, pH 7.4) or extracellular Ca^2+^-free solution containing 5 mM EGTA and no CaCl_2_. Intracellular Ca^2+^ concentrations in individual cells were determined by the ratio of F340/F380, the fluorescence intensities that excited alternatively at 340 and 380 nm, emitted at 510 nm, and captured using an Axiovert S100TV microscope (Zeiss) and Openlab 2 software (Image Processing and Vision, Agilent, Santa Clara, CA, USA). Extracellular solutions alone or containing 300 μM H_2_O_2_ were applied to cells by a gravity-fed perfusion system after establishment of the baseline. In some experiments, cells were initially exposed to H_2_O_2_ in a Ca^2+^-free solution and then in Ca^2+^-containing solutions. F340/F380 values in each cell were normalized to the basal value at the time of starting H_2_O_2_ application.

### 2.6. Cell Death Assay

Cells were seeded in a 96-well plate at a concentration of 7 × 10^3^ cells/mL and incubated overnight at 37 °C before use. Cells were treated with indicated concentrations of H_2_O_2_ or EtOH at 37 °C for 24 h, in the majority of experiments, or EtOH for 8 h, in a small number of experiments, as specifically indicated in figure legends. After treatment with H_2_O_2_ or EtOH, cells were incubated with 2 μg/mL propidium iodide (PI) at 37 °C for a further 30 min prior to imaging. Cells were co-stained with 5 μg/mL Hoechst 33342 (Hoechst) or 1 μg/mL acridine orange (AO). Cells were imaged using a fluorescent microscope equipped with a camera (Nikon). The number of PI-positive dead cells and the total number of cells identified by co-staining with Hoechst or AO in three randomly-chosen areas in each image were counted using ImageJ, and at least 80 cells were examined in each well. Cell death was presented by expressing PI-positive cells as a percentage of all cells identified in the same areas.

### 2.7. Measurement of ROS Production

Intracellular ROS production was assessed using 2′,7′-dichlorodihydrofluorescein diacetate (DCFH-DA) (Sigma) by measuring the 2’,7’-dichlorofluorescein (DCF) fluorescence intensity, as described in our previous study [31]. Briefly, cells plated in 96-well plates were treated with EtOH at indicated concentrations for 8 h. Cells were washed with PBS before being loaded with 20 µM DCFH-DA in PBS at 37 °C for 20 min. Cells were washed with PBS prior to imaging. Images were captured using a fluorescent microscope (Nikon) and NIS-Elements Viewer software. The DCF fluorescent intensity in each cell was quantified using ImageJ and at least 75 cells were examined in each well.

### 2.8. Data Presentation and Statistical Analysis

All data are presented as mean ± standard error of mean, obtained from individual cells for single-cell calcium imaging or each independent cell preparation for cell death and ROS production. Statistical analysis was performed using the Student’s *t*-test for comparisons of two groups and a one-way ANOVA followed by post hoc Tukey’s test for comparison among multiple groups, with *p* < 0.05 being statistically significant.

## 3. Results

### 3.1. Expression of TRPM2 in Microglial Cells and Its Role in H_2_O_2_-Induced Cell Death

**Figure 1:**
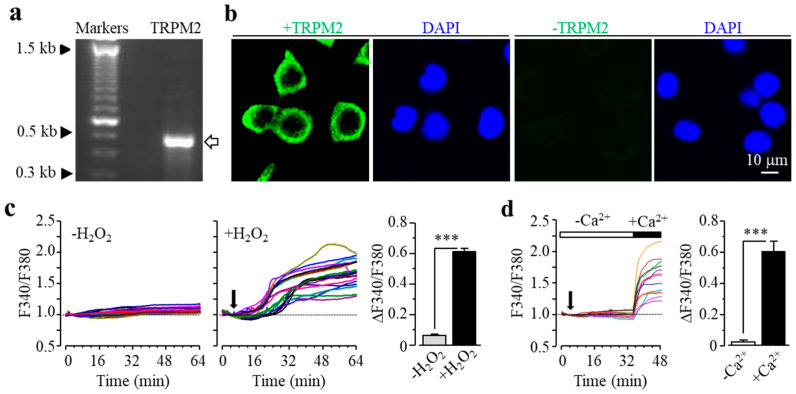
Transient receptor potential melastatin-related 2 (TRPM2) expression in BV2 microglial cells. (**a**) Agarose gel analysis showing TRPM2 mRNA expression (the arrow denotes the PCR product with the expected size of 479 bp). (**b**) Representative confocal images showing cells stained with the TRPM2 antibody and 4’,6-diamidino-2-phenylindole (DAPI) (left) or stained only with the second antibody and DAPI (right). (**c**) Left: F340/F380 in individual cells without or with exposure to 300 μM H_2_O_2_ (indicated by the downward arrow). Right: mean change in F340/F380 after 30 min exposure to H_2_O_2_ or equivalent time point (63 control cells and 78 H_2_O_2_-exposued cells). (**d**) Left: F340/F380 in individual cells during exposure to 300 μM H_2_O_2_, firstly in extracellular Ca^2+^-free solution and then Ca^2+^-containing solution, indicated by the open and the solid bars above, respectively. Right: mean change in F340/F380 during exposure to H_2_O_2_ in Ca^2+^-free and Ca^2+^-containing solutions (14 cells). *** *p* < 0.005 compared to indicated group.

**Figure 2:**
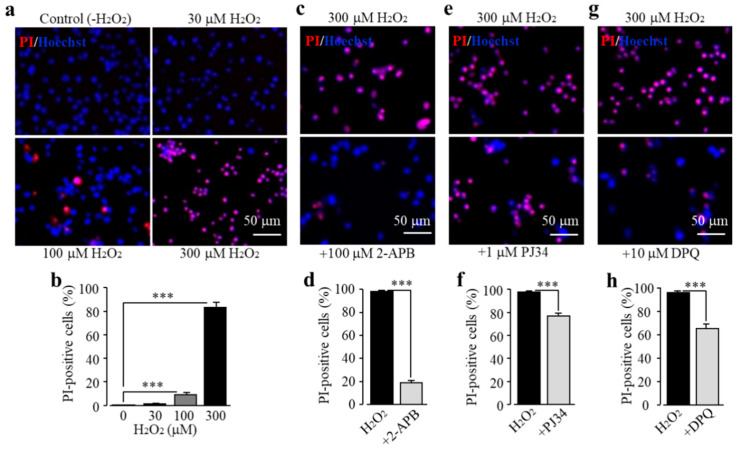
Reactive oxygen species (ROS) induce BV2 microglial cell death via poly(ADP-ribose) polymerase (PARP)-dependent TRPM2 channel activation. (**a**,**b**) Representative fluorescent images showing co-staining with propidium iodide (PI) and Hoechst (**a**) and mean percentage of PI-positive cells (**b**) in cells without (control) and with exposure to indicated concentrations of H_2_O_2_ for 24 h. (**c**–**h**) Representative fluorescent images showing co-staining with PI and Hoechst in cells exposed to 300 μM H_2_O_2_ for 24 h without and with treatment with indicated inhibitors (**c**,**e**,**g**) and mean percentage of PI-positive cells (**d**,**f**,**h**). Cells were pre-treated with inhibitors 30 min prior to and during exposure to H_2_O_2_. Mean data are from at least three independent experiments, using three wells of cells for each condition in each experiment. *** *p* < 0.005 compared to indicated group.

We characterized TRPM2 channel expression in BV2 microglial cells using RT-PCR and immunofluorescent imaging. TRPM2 mRNA and protein expression was readily detected (Figure 1a,b). Of note, TRPM2 immunoreactivity was highly concentrated on or in the vicinity of the plasma membrane (Figure 1b). As shown using single-cell imaging, individual cells responded to exposure to H_2_O_2_ (300 μM), a widely used paradigm of inducing cellular oxidative stress, with a salient increase in intracellular Ca^2+^ concentration (Figure 1c). Furthermore, single-cell imaging using the Ca^2+^ add-back protocol revealed that such robust Ca^2+^ responses induced by exposure to H_2_O_2_ resulted from extracellular Ca^2+^ influx (Figure 1d). Taken gather, these data suggest that the TRPM2 channel mainly functions as a Ca^2+^-permeable channel on the cell surface, as reported in primary microglial cells [28,29,30]. We further examined whether prolonged exposure to ROS induced cell death via the TRPM2 channel. There were very few PI-positive dead cells under the control condition, but the percentage of PI-positive cells was significantly increased following exposure to 100–300 μM H_2_O_2_ (Figure 2a,b). Such cell death was significantly inhibited by treatment with 2-aminoethoxydiphenyl borate (2-APB), a known TRPM2 channel inhibitor (Figure 2c,d), or by treatment with PJ34 and 3,4-dihydro-5[4-(1-piperindinyl)butoxy]-1(2H)-isoquinoline (DPQ), two structurally different PARP inhibitors (Figure 2e–h). Thus, exposure to oxidative stress can induce PARP-dependent TRPM2 channel activation in BV2 microglial cells that can lead to cell death, highly consistent with our recent study examining primary microglial cells [25].

### 3.2. Exposure to EtOH Induces Microglial Cell Death via PARP-Dependent TRPM2 Channel Activation

**Figure 3:**
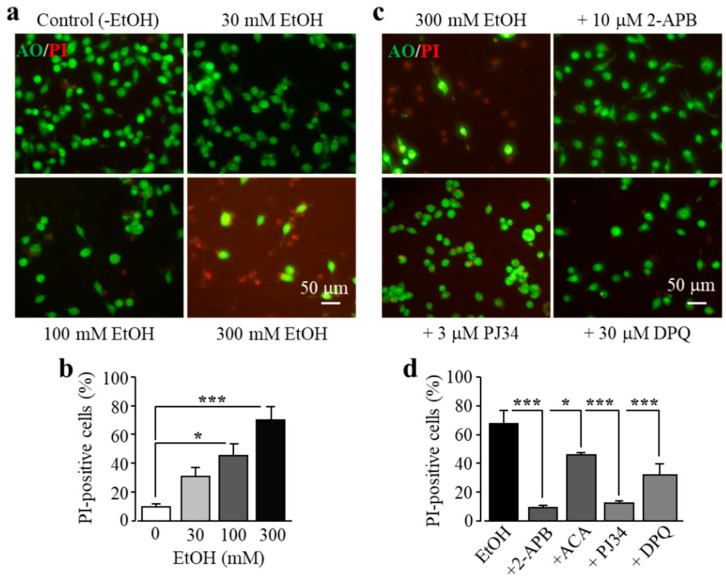
Ethanol (EtOH) induces BV2 microglial cell death via PARP-dependent TRPM2 channel activation. (**a**,**b**) Representative fluorescent images showing co-staining with PI and acridine orange (AO) (**a**) and mean percentage of PI-positive cells (**b**) in cells without (control) and with exposure to indicated concentrations of EtOH for 24 h. (**c**,**d**) Representative fluorescent images showing co-staining with PI and AO in cells exposed to 300 mM EtOH for 24 h without and with treatment with indicated inhibitors (**c**) and mean percentage of PI-positive cells from three independent experiments, using three wells of cells for each condition in each experiment (**d**). Cells were pre-treated with inhibitors 30 min prior to and during exposure to EtOH. * *p* < 0.05; *** *p* < 0.005 compared to indicated control group.

As introduced above, exposure to high doses of alcohol can induce ROS generation and oxidative stress, but it is unknown whether alcohol-induced oxidative stress can induce cell death in microglial cells. Therefore, we investigated the effects of exposure to EtOH for 24 h at concentrations (10–300 mM) which have been commonly used for in vitro studies [34,35,37,38,39]. As shown in Figure 3a,b, exposure to EtOH induced concentration-dependent microglial cell death, with the cell death level significantly increased following exposure to high concentrations (100 and 300 mM). As shown above for H_2_O_2_-induced cell death, EtOH-induced cell death was also strongly attenuated by treatment with 2-APB and N-(p-amylcinnamoyl)anthranilic acid (ACA), another TRPM2 channel inhibitor, or by treatment with PJ34 and DPQ (Figure 3c,d). These results are consistent with the notion that EtOH induces microglial cell death via PARP-dependent TRPM2 channel activation.

### 3.3. EtOH-Induced Microglial Cell Death Depends on NOX-Mediated ROS Generation

**Figure 4:**
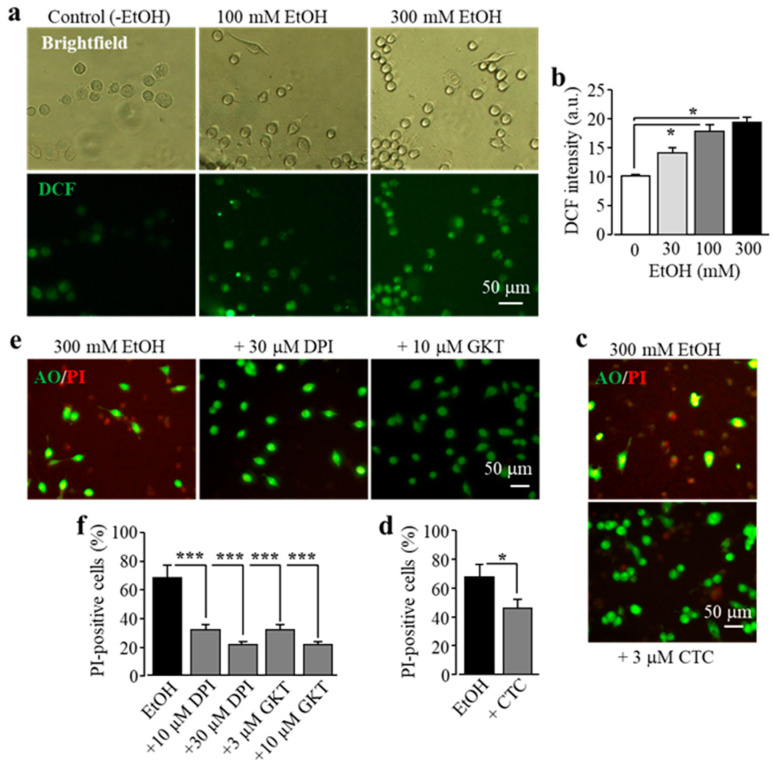
EtOH induces cell death in BV2 microglial cells via protein kinase C (PKC) and NOX-mediated ROS production. (**a**,**b**) Representative fluorescent images showing cellular ROS level (top row: brightfield in phase-contrast; bottom row: 2’,7’-dichlorofluorescein (DCF ) fluorescence) in microglial cells without (control) and with exposure to indicated concentrations of EtOH for 8 h (**a**), and mean DCF fluorescence intensity in microglial cells under indicated conditions from three independent experiments, using three wells of cells for each condition in each experiment. (**c**–**f**) Representative fluorescent images showing co-staining with PI and AO (**c**,**e**) and mean percentage of PI-positive cells (**d**,**f**) in cells without (control) and with exposure to indicated concentrations of EtOH for 8 h alone or together with chelerythrine chloride (CTC), diphenyleneiodonium (DPI), or GKT -137831 (GKT). Cells were pre-treated with the inhibitors 30 min prior to and during exposure to EtOH. Mean data are from three independent experiments, using three wells of cells for each condition in each experiment. * *p* < 0.05; *** *p* < 0.005 compared to indicated control group.

It is known that exposure to alcohol induces ROS generation via NOX [11]. Previous studies by our group and other authors showed that activation of NOX is crucial in Zn^2+^-induced ROS production in neurons and microglial cells, with a dependency of protein kinase C (PKC) in microglia [25,31,40]. Therefore we hypothesized that PKC and NOX play a similar role in EtOH-induced ROS production that induces subsequent activation of PARP and the TRPM2 channel, leading to microglial cell death. To address this, we firstly used single-cell imaging to measure the intensity of DCF, a fluorescent indicator for ROS generation, to investigate whether exposure to EtOH promoted ROS production in BV2 microglial cells. Exposure to 30–300 mM EtOH induced a concentration-dependent increase in the cytosolic ROS level, with the level being significantly higher after exposure to 100 and 300 mM (Figure 4a,b), consistent with the fact that NOX mediates alcohol-induced ROS generation. EtOH-induced microglial cell death was strongly attenuated by treatment with CTC, a PKC inhibitor (Figure 4c,d), and also by treatment with diphenyleneiodonium (DPI), a NOX generic inhibitor, and by treatment with GKT-137831, a NOX1/4-specific inhibitor (Figure 4e,f). Collectively, these data support a significant role of NOX-mediated ROS generation in ethanol-induced TRPM2 channel activation and cell death.

## 4. Discussion

**Figure 5:**
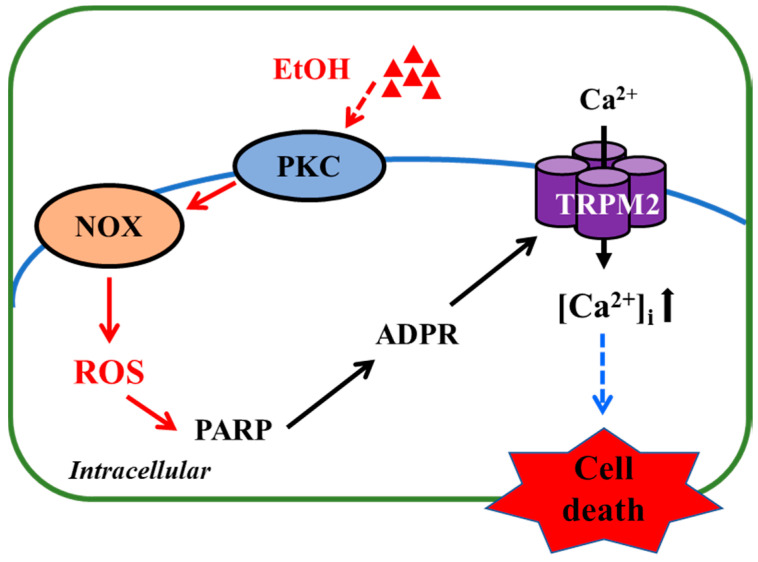
TRPM2-mediated signaling mechanism for EtOH-induced microglial cell death. Exposure to EtOH stimulates PKC and NOX to generate ROS, which, in turn, induce PARP-dependent ADP-ribose (ADPR) production and subsequent TRPM2 channel activation, leading to an increase in intracellular Ca^2+^ concentrations ([Ca^2+^]_i_) and cell death. Abbreviations: PKC, protein kinase C; NOX, nicotinamide adenine dinucleotide phosphate oxidase; ROS, reactive oxygen species; PARP, poly (ADP-ribose) polymerase; ADPR, ADP-ribose.

In the present study, we show that exposure to high concentrations of EtOH induced microglial cell death and provide evidence to suggest that EtOH-induced microglial cell death occurs via the NOX/ROS/PARP/TRPM2 signaling pathway (Figure 5).

It is well documented that excessive consumption of alcohol has extensive harmful effects on the brain among many other parts of the human body [1,6], and how exactly alcohol affects the brain remains a hot research topic. As introduced above, an increasing number of studies support a significant role of microglial cells in alcohol-induced brain pathologies [7,11,16,17,18,19,20,21,22,23,24]. However, the mechanisms driving the progression of microglial cell-associated alcohol pathologies still remain not fully understood. There is a large body of evidence suggesting a crucial role of the TRPM2 channel in cell death in diverse cell types, including microglial cells, induced by exposure to ROS or by pathological factors that are known to stimulate ROS generation [25,26,27,28,29,31,33]. However, it was not clearly defined whether the TRPM2 channel is important in EtOH-induced microglial cell death. In the current study, using BV2 microglial cells, we firstly demonstrated that the TRPM2 channel is expressed in BV2 microglial cells (Figure 1) and mediates H_2_O_2_-induced cell death (Figure 2a,b). These observations are highly consistent with what we have recently reported in primary microglial cells [25]. Like exposure to H_2_O_2_, we showed that exposure to EtOH evoked significant microglial cell death (Figure 3a,b). Furthermore, similar to H_2_O_2_-induced cell death (Figure 2c), EtOH-induced cell death was strongly inhibited by treatment with 2-APB and, to a lesser extent, ACA, two structurally different TRPM2 channel inhibitors (Figure 3c,d). These results, together, support a crucial role of the TRPM2 channel in mediating EtOH-induced microglial cell death. It is known that exposure to alcohol leads to oxidative stress and activation of PARP [8,9,10] and, as introduced earlier, PARP activation is the major signaling mechanism for oxidative stress-induced TRPM2 channel activation, including in microglial cells [25,26,27,28,29,31,32,33]. As described in our recent study examining cell death induced by ROS or Zn^2+^ in primary microglial cells [25], and in this study showing H_2_O_2_-induced cell death in BV2 microglial cells (Figure 2e–h), EtOH-induced microglial cell death was remarkably attenuated by treatment with PJ34 and DPQ, two structurally different PARP inhibitors (Figure 3), supporting that PARP activation is critical in EtOH-induced TRPM2-mediated microglial cell death. Taken together, these results suggest that exposure to EtOH induces microglial cell death via PARP-dependent TRPM2 channel activation.

In the current study, we also investigated the signaling mechanisms by which EtOH stimulates activation of PARP and the TRPM2 channel, which leads to subsequent cell death in microglial cells. ROS production is one of the common events involved in mediating TRPM2 channel activation [26,27,28,29]. It has been well documented that EtOH can stimulate ROS production [9] via induction of NOX [11]. Consistent with this notion, we showed that exposure to high concentrations of EtOH induced a significant increase in ROS production in microglial cells (Figure 4a,b) and, furthermore, that EtOH-induced microglial cell death was prevented by treatment with PKC and NOX inhibitors (Figure 4c–f), consistent with the notion that PKC/NOX-mediated ROS production acts as an upstream mechanism in mediating EtOH-induced TRPM2 channel activation and microglial cell death.

To summarize, the results presented in this study suggest that exposure to EtOH at pathologically relevant concentrations results in microglial cell death via induction of NOX-mediated ROS generation and subsequent activation of PARP and the TRPM2 channel. As illustrated in Figure 5, such a mechanism offers novel and potentially important insights into alcohol-induced brain pathologies. Nevertheless, it is evident that more investigations are warranted to explore this new mechanism. In microglial cells, the TRPM2 channel is mainly expressed on the cell surface and plays a key role in mediating ROS-induced Ca^2+^ influx [32,33], which is endorsed with the results we obtained in BV2 microglial cells (Figure 1c,d), but the role of such TRPM2-mediated Ca^2+^ signalling in EtOH-induced microglial cell death remains to be ascertained. We have recently shown that H_2_O_2_/Zn^2+^-induced cell death in primary microglial cells occurs predominantly via necrosis [25], and it is interesting to establish whether such a mechanism also underpins EtOH-induced microglial cell death. Most importantly, in vivo studies using rodent models of alcohol-induced brain pathologies in combination with pharmacological or genetic interventions are needed to testify the significance of the finding reported in this study.

## 5. Conclusions

Our study shows that exposure to EtOH induces microglial cell death via the NOX/ROS/PARP/TRPM2 signaling pathway. Our finding provides novel insights into the mechanisms potentially underlying alcohol-induced brain pathologies and suggests targeting the TRPM2 channel as an avenue to treat such conditions.
